# Sabine Roeser, Rafaela Hillerbrand, Per Sandin, Martin Peterson (eds): Handbook of Risk Theory: Epistemology, Decision Theory, Ethics, and Social Implications of Risk: Springer, 2012, XIX, 1187 pp, 85 illus., 40 in color; 2 volumes

**DOI:** 10.1007/s11948-012-9362-y

**Published:** 2012-04-26

**Authors:** Neelke Doorn

**Affiliations:** Department of Technology, Policy and Management, 3TU, Centre for Ethics and Technology, Delft University of Technology, PO Box 5015, 2600 GA Delft, The Netherlands

Risk is a central notion in a broad range of fields, varying from engineering to medicine, from life sciences to economics. It is also a topic being studied from different perspectives and by people with different disciplinary backgrounds. This raises not only questions related to the nature of risk—a question which may be answered differently by different people—but also fundamental questions related to the study of risks, risk perception, risk and society, and the acceptability of risks. These and other questions related to risks could be captured under the general heading of risk theory. Sabine Roeser, together with co-editors Rafaela Hillerbrand, Per Sandin and Martin Peterson, has compiled a comprehensive handbook of risk theory, addressing many of the topics mentioned above.

The *Handbook of Risk Theory* aims at providing an overview into the key topics in risk theory, ranging from epistemology, decision theory, risk perception, to ethics and the social implications of risks. In addition, some specific case studies are discussed in order to identify specific risks. With all four editors having a background in philosophy, the unique philosophical angle to risk theory does not come as surprise.

The handbook is divided into six parts: general issues in risk theory (Part 1), specific risks (Part 2), decision theory and risk (Part 3), risk perception (Part 4), risk ethics (Part 5), and risk in society (Part 6). Together, these six parts cover as many as 46 chapters, written by philosophers and other scholars working on risk theory. The editors have chosen not to write a substantive introduction. Apart from a brief introduction to the six parts, the introduction is primarily a brief overview of the respective chapters. Neither is there a concluding section at the end of the book. Given that the book is meant as a handbook rather than an edited volume, this choice is understandable.

Following the introduction by the editors, Sven Ove Hansson, one of the leading philosophers in the field of risk theory, sketches a panorama of the philosophy of risks (Chapter 2). This is followed by a contribution on the concepts of risk and safety (Niklas Möller; Chapter 3) and one of levels of uncertainty (Hauke Riesch; Chapter 4). Together, these contributions provide a firm basis for the book.

In Part 2, specific risks are discussed. The aim of this part is to show how real natural or anthropogenic hazards bring about the necessity to reflect on risks. This part addresses natural, technological, and societal risks from the perspective of the natural and social sciences by also incorporating insights from the humanities and mathematical sciences. One of the outstanding contributions in this part is the one by Louis Eeckhoudt and Henri Loubergé on the economics of risk (Chapter 5). In this chapter, the authors provide a brief overview of the treatment of risk in economics, starting from early principles in the 1940s and 1950s and extending until the most recent developments. This chapter clearly illustrates what role the notion of risk plays and has played in economics.

Part 3 is dedicated to decision theory and risk. In their introduction, the editors explain how they use the term “decision theory,” viz. the theory of rational decision making about risks, or, in other words, the study of normative hypotheses about how it would be rational to take risky decisions. In the introduction, the editors describe the mainstream view in risk theory as linking rational decision making to maximizing expected utility. This principle “takes the total value of an act to equal the sum total of the values of its possible outcomes weighted by the probability for each outcome. The values assigned to an outcome are determined by the decision maker’s desires, whereas the probabilities are determined by his or her beliefs about how likely the outcomes are to materialize” (p. 9).

In nine different chapters, the various aspects of decision theory in the context of risk are discussed, including some problems it prompts. Not surprisingly, the contributions in this part are somewhat more technical than those in the other parts. However, they provide a very valuable introduction to decision theory in relation to risks, and as such they are indispensable for the book.

Risk perception is the overarching theme of Part 4. This is, what the editors call, the descriptive counterpart of the normative discussion in decision theory. In this part, empirical claims about how people actually make risky decisions are discussed. This part draws heavily on psychological and anthropological insights, like the work of Paul Slovic (2000) and Mary Douglas (Douglas 1985; Douglas and Wildavsky 1982). From a normative point of view, this part is somewhat problematic. Risk communication is controversial if it only serves to get support for some “risky decision.” Uncritical use of insights from communication sciences is therefore dubious at least. Hence, risk communication brings along moral questions that deserve reflection on their own. In neither of the contributions is this aspect explored or even mentioned, which is an unfortunate omission.

Ethics is the central topic of Part 5, aptly entitled risk ethics. It is by now increasingly recognized by risk scholars that “risk is not a purely quantitative notion but also involves qualitative, normative, and ethical considerations” (p. 16). In this part, the aspects of risks that require ethical scrutiny are identified, such as the relation between risk and trust, responsibility, justice, criteria for assessing the moral acceptability of risks, use of the precautionary principle etc. Some of these aspects have been covered elsewhere (for example, in the book *The Ethics of Technological Risks*, which the editor-in-chief of the current handbook published with Earthscan (Asveld and Roeser 2009), but the current contributions provide a valuable update of that volume.

The part on risk ethics provides a prelude to the concluding Part 6, Risk in Society. In this part, the implications of risk for people’s lives are discussed. Topics like risk management and risk governance are discussed. In this part, the relatively recent activities of risk analysis, risk management, and risk governance are placed in historical perspective. This part links the theory outlined in the previous parts to practice, where actual decisions need to be made. The editors quote Ulrich Beck’s interpretation of “risk society” (Beck 1992), when they describe this part as discussing “the ways society does and should cope with risk” (p. 20). With some very interesting contributions from scholars working for governmental advisory boards and councils (e.g., the two contributions co-written by Van Asselt on risk governance (Chapter 44) and EU risk regulation and the uncertainty challenge (Chapter 45), respectively), this part is indispensable for realizing the ambition of the editors, viz. to show that “when it comes to risk, theory and practice are closely intertwined” (p. 23). The main body of the book is dedicated to the theory. However, with the last part on risk in society, this link to practice is clearly made.

All in all, the editors have managed to compile an excellent handbook with contributions by leading scholars in risk theory. Although aimed at experts with a scientific background in risk, many of the book chapters would not be misplaced in (post)graduate courses on risk theory. In addition to the content, the book is carefully edited, with a brief table of content and list of references for each of the chapters, making the chapters also easily accessible on an individual basis.

## References

[CR1] Asveld L, Roeser S (2009). The ethics of technological risk.

[CR2] Beck U (1992). Risk society: towards a new modernity.

[CR3] Douglas M (1985). Risk acceptability according to the social sciences.

[CR4] Douglas M, Wildavsky A (1982). Risk and culture.

[CR5] Slovic P (2000). The perception of risk.

